# Opportunities and challenges of self-binding directives: an interview study with mental health service users and professionals in the Netherlands

**DOI:** 10.1186/s12910-023-00915-y

**Published:** 2023-06-03

**Authors:** Laura van Melle, Lia van der Ham, Yolande Voskes, Guy Widdershoven, Matthé Scholten

**Affiliations:** 1 Department of Ethics, Law and Humanities, Amsterdam UMC, Vrije Universiteit Amsterdam, Amsterdam, The Netherlands; 2 Institute for Medical Ethics and History of Medicine, Ruhr University Bochum, Markstr. 258a, 44799 Bochum, Germany

**Keywords:** Self-binding directive, Ulysses contract, Ulysses arrangement, Psychiatric advance directive, Advance statement, Crisis plan, Mental healthcare, Psychiatry, Coercion

## Abstract

**Background:**

Self-binding directives (SBDs) are psychiatric advance directives that include the possibility for service users to consent in advance to compulsory care in future mental health crises. Legal provisions for SBDs exist in the Netherlands since 2008 and were updated in 2020. While ethicists and legal scholars have identified several benefits and risks of SBDs, few data on stakeholder perspectives on SBDs are available.

**Aims:**

The aim of the study was to identify opportunities and challenges of SBDs perceived by stakeholders who have personal or professional experience with legally enforceable SBDs.

**Methods:**

Data collection was carried out in the Netherlands from February 2020 to October 2021 by means of semi-structured interviews. Participants were selected through purposive sampling and snowball methods. Interviews were conducted with mental health service users (n = 7), professionals (n = 13), and an expert on SBD policy (n = 1), resulting in a total number of 21 interviews. The data were analyzed thematically.

**Results:**

Perceived benefits of SBDs included increased autonomy, improvement of the therapeutic relationship, possibility of early intervention and prevention of harm, prevention of compulsory care, reduction of the duration of compulsory care and recovery, mitigation of negative experiences around compulsory care, and guidance for professionals in providing compulsory care. Perceived risks included infeasibility of SBD instructions, difficulty in decision-making around SBD activation, limited accessibility of SBDs, disappointment of service users due to non-compliance with SBDs, and limited evaluation and updating of SBD content. Barriers to SBD completion included lack of knowledge of SBDs among professionals, lack of motivation or insight among service users, and lack of professional support for SBD completion. Facilitators of SBD completion and activation included support for SBD completion, involvement of relatives and peer experts, specification of SBD content, and evaluation of compulsory care and SBD content. The new legal framework was regarded as having both positive and negative effects on SBD implementation.

**Conclusions:**

Stakeholders who have personal or professional experience with legally enforceable SBDs perceive SBDs as having important benefits and tend not to articulate the fundamental ethical concerns about SBDs which can be found in the ethics and legal literature. Instead, they perceive ethical and practical challenges that can be addressed through the implementation of suitable safeguards.

**Supplementary Information:**

The online version contains supplementary material available at 10.1186/s12910-023-00915-y.

## Background

Self-binding directives (SBDs) are psychiatric advance directives that include the possibility for mental health service users to consent in advance to compulsory care in future mental health crises [1]. They are also commonly referred to as Ulysses contracts or Ulysses arrangements, referring to Homer’s Ulysses, who on his journey home to Ithaca managed to relish hearing the enticing song of the Sirens without it being his downfall by instructing his crew to fasten him to the mast of the ship and ignore his pleas to be liberated [2–5]. SBDs can be helpful for people with mental disorders that entail fluctuating mental capacity and a high likelihood of treatment refusals during crisis, such as bipolar and psychotic disorders [6]. During manic or psychotic episodes, people sometimes show harmful behavior that is incongruent with their deeply-held values and beliefs while refusing hospital admission and treatment. By enabling service users to plan compulsory care in advance and to instruct professionals to overrule such treatment refusals, SBDs can help service users to maintain control over their life and treatment.

The Netherlands is one of the very few jurisdictions worldwide with legal provisions for SBDs [7, 8]. Although SBD regulation exists since 2008, the implementation of SBDs progresses very slowly: completion rates have remained very low and compulsory care is rarely provided based on an SBD [7]. This is presumably due to known barriers to the completion of psychiatric advance directives, such as lack of knowledge and awareness about the instrument among service users and professionals, lack of training and guidance for professionals, and lack of support for SBD completion [9]. Another barrier to the implementation of SBDs is the complexity of the Dutch SBD regulation. On the 1st of January 2020, the Dutch Law on Compulsory Mental Health Care (*Wet verplichte geestelijke gezondheidszorg*; *Wvggz*) entered into force, resulting in a new legal framework for SBDs. Within the new legal framework, obtaining legal authorization for providing compulsory care based on an SBD remains subject to highly complex and lengthy formal procedures [8]. These barriers restrict access to SBDs by service users and keep SBDs from reaching their full potential of giving service users increased control over their life and treatment.

The use of SBDs in mental healthcare is controversial and has sparked intense debates among philosophers, medical ethicists, and legal scholars. In these debates, several potential benefits and risks of SBDs have been identified by means of conceptual, legal, and ethical analysis. Proponents of SBDs have identified the following benefits of SBDs: promotion of service user autonomy and wellbeing, facilitation of early intervention, improvement of relationships with professionals and informal caregivers, reduction of perceived coercion, and relief of the burden on substitute decision-makers [5–7, 10–14]. Critics of SBDs have identified the following risks of SBDs: self-paternalism, susceptibility to undue influence during the drafting process, increase of coercion due to premature SBD activation, the impossibility to accomodate for changes of mind, and expired consent [3, 4, 15, 16].

Few empirical studies on SBDs have been conducted to date. Systematic reviews on advance care planning in mental healthcare reported a high interest among people with bipolar and psychotic disorders in psychiatric advance directives [17] and SBDs in particular [18]. In a survey among persons with bipolar disorder (N = 932), 69% of respondents were in favor of having a self-binding clause in their psychiatric advance directive [19]. An analysis of free text answers of respondents to this survey (N = 565) revealed that 82% were in favor of SBDs, 7% was ambivalent, and 12% rejected SBDs. Those who endorsed SBDs predominantly cited distorted thinking during mental health crises as a reason, while those who were ambivalent or rejected SBDs predominantly cited reasons related to practical implementation issues [20].

Rosenson et al. [21] conducted an informal survey among service users (n = 9), relatives (n = 12), and professionals (n = 8). A recurring theme among respondents was the need for safeguards. Proposed safeguards included the involvement of a third party in the drafting process, administration of compulsory care by the professional who signed the SBD, and hearings to authorize compulsory care based on an SBD. Stephenson et al. [22] evaluated an SBD template in focus groups with service users (n = 10), relatives (n = 3), and professionals (n = 19) in the UK, and Potthoff et al. [23] conducted one focus group with researchers (n = 5) and interviews with service users (n = 6), relatives (n = 6), and professionals (n = 5) in Germany. Benefits of SBDs reported in these studies included promotion of autonomy, improvement of the therapeutic relationship, and avoidance of harm. Risks included undue influence during SBD completion, risk of misinterpretation of SBD content, and limited flexibility in medical decision-making due to narrow SBD instructions. None of these studies was conducted in a context where SBDs were legally enforceable, implying that stakeholders were hypothetically considering SBDs without having personal or professional experience with the instrument.

Two stakeholder studies on SBDs have been conducted in the Netherlands. Varekamp [24] carried out interviews with service users (n = 18) and professionals (n = 17), and Gremmen et al. [12] carried out interviews with service users (n = 18), relatives (n = 12), professionals (n = 17), and other parties involved (n = 19). Both these studies were carried out before provisions for SBDs were included in the Dutch law and in neither of the studies having personal or professional experience with SBDs was an inclusion criterion, though Varekamp somewhat vaguely reports that “six clients did have a kind of Ulysses directive” [24]. Key benefits of SBDs reported in these studies included the possibility of timely intervention, the avoidance of harm, and the promotion of autonomy. Key challenges included undue influence during SBD completion, and premature hospital admission.

The primary aim of our study was to identify the benefits and risks of SBDs perceived by stakeholders who have personal or professional experience with legally enforceable SBDs. The secondary aim was to explore stakeholders’ perspectives on the barriers and facilitators related to SBD implementation and the effects of the Dutch Law on Compulsory Mental Health Care on SBD implementation. To the best of our knowledge, this is the first SBD study worldwide with stakeholders who have personal or professional experience with legally enforceable SBDs.

## Methods

### Design

We chose a qualitative research design using semi-structured interviews and thematic data analysis to gain in-depth insight into stakeholders’ perspectives on SBDs. The researchers involved in the study have backgrounds in medical ethics, mental health nursing, philosophy, and psychology. The study was carried out from February 2020 to October 2021 in the Netherlands. The COREQ checklist was used as guidance for reporting [25].

### Sampling method and participants

The inclusion criteria for the study were having an SBD (for service users) and treating service users who have an SBD (for professionals). We contacted psychiatrists from four large mental health institutions in three different regions in the Netherlands by e-mail. We selected them based on their experience with SBDs. These participants referred us to other professionals in their organization who had experience with SBDs. We included thirteen mental health care professionals, including psychiatrists, psychologists, nurses, and a nurse practitioner. We also interviewed one expert on SBD policy. We found service users who have an SBD via the professionals who participated in the study. After having interviewed a professional, we asked them to forward the call for participation to service users who have an SBD. The service users included in the study contacted us of their own accord. We were able to include seven service users, of which three worked as a peer expert at a mental health institution. Peer experts are people with lived experience who are employed by a mental health institution and who are trained to give guidance to service users during treatment. We explicitly asked service users who worked as peer experts to share their experiences as a service user. No participants dropped out during the research process. Participants varied by gender (10 female and 11 male) and profession. The characteristics of participants are summarized in Table 1.

**Table 1:** Sample characteristics

Nr.	Discipline	Experience with SBDs
1	Service user and peer expert	Has had an SBD for 12 years
2	Service user	Has had an SBD for 1 year
3	Service user and peer expert	Has had an SBD for several years
4	Service user	Has had an SBD since recently
5	Service user	Has had an SBD for 13 years
6	Service user	Has had an SBD for 6 months
7	Service user and peer expert	Has had an SBD for 1 year
8	Psychiatrist and medical director	Experience in reviewing and approving SBDs
9	Psychiatrist and medical director	Has drafted SBDs with 4 service users, the first of which 6 years ago
10	Psychiatrist and medical director	Experience in reviewing and approving SBDs
11	Psychiatrist	Has drafted SBDs with 4 service users
12	Psychiatrist	Has drafted an SBD with a service user 10 years ago
13	Psychiatrist	Has drafted SBDs with 2 service users
14	Psychiatrist	Has drafted an SBD with a service user and has experience with mental capacity assessment in the context of SBD completion
15	Psychiatrist	Has drafted multiple SBDs with service users
16	Nurse practitioner	Has drafted SBDs with several service users
17	Psychologist	Has treated several service users with an SBD
18	Psychologist	Has treated a service user with an SBD during and after hospital admission
19	Community mental health nurse	Has drafted an SBD with a service user 1 year ago
20	Clinical mental health nurse	Has drafted an SBD with a service user
21	Policy expert	Scientific and legal knowledge of SBDs

### Data collection

Semi-structured interviews were carried out using an interview guide (see online supplementary materials). We developed the interview guide based on the preliminary results of a review of the conceptual literature on SBDs and discussions among MS and LvM. Main topics in the guide were procedures for SBD completion and evaluation, benefits and risks of SBDs, barriers and facilitators related to SBD implementation, and the effects of the new law on SBD implementation. LvM and MS jointly carried out the first interview and LvM carried out the further interviews. Both researchers had experience in conducting interviews. Interviews lasted approximately 40–60 min. We carried out eight interviews at a location of convenience for the participant and thirteen interviews online by means of Zoom or Google Meet due to COVID-related restrictions. Data collection was continued until data saturation was reached. All interviews were audiotaped, transcribed verbatim, and pseudonymized. Transcripts of the interviews were not returned to participants for comments and corrections.

### Data analysis

We analyzed the interview data based on the Dutch transcripts using thematic analysis according to Braun and Clarke [26] and the software MAXQDA version 2018. We started the data analysis during data collection and combined a deductive and inductive coding approach. MS developed an initial coding scheme based on his knowledge of the literature on SBDs and adjusted it in consultation with LvM and LvdH, who had familiarized themselves with the data. LvM and LvdH separately coded the transcripts of two interviews each by using the initial coding scheme and expanded and adjusted this coding scheme inductively. They compared coded segments and emerging codes, and discussed discrepancies with MS until consensus was reached. The resulting coding scheme provided the basis for the analysis of the remaining transcripts. LvM, LvdH and MS discussed the final themes and subthemes with GW and YV, who were not involved in data collection and the first analysis, to ensure objectivity and to strengthen the analysis. Interview excerpts cited in this article were translated into English after the data analysis was concluded.

### Ethical considerations

Before commencing the interview, we orally informed participants about the details of the study and their right to withdraw their consent and have their data deleted until publication of the data. We also provided participants with an information leaflet and asked them to give written consent. Participants who were interviewed online gave verbal consent and their consent was recorded. The Medical Ethical Review Committee of Amsterdam University Medical Center, registered with the US Office for Human Research Protections (OHRP) as IRB00002991, reviewed the study protocol, patient information and informed consent leaflet of the study, and declared that the study does not fall under the scope of the Dutch Medical Research Involving Human Subjects Act (WMO) and hence is not subject to official approval by the committee (reg. no. 2019.730).

## Results

Following our research questions and interview guide, the main themes were benefits and risks of SBDs, barriers and facilitators related to SBD completion and activation, and the effects of the new law on the implementation of SBDs. For each of these themes, various subthemes emerged from the data. These are summarized in Table 2 and described below.

**Table 2:** Overview of results

Benefits	Risks
increased service user autonomy	infeasibility of SBDs
improvement of the therapeutic relationship	difficulty in decision-making about SBD activation
possibility of timely intervention and prevention of harm	limited accessibility of SBDs in mental health crises
prevention of compulsory care	disappointment of service users due to professionals’ non-compliance with SBDs
reduction of the duration of compulsory care and recovery	limited evaluation and updates of SBD content
mitigation of negative experiences around compulsory care	
guidance for professionals in providing compulsory care	
**Facilitators**	**Barriers**
support for SBD completion	lack of knowledge of SBDs among professionals
involvement of relatives and peer experts	lack of motivation or insight among service users
specification of SBD content	lack of professional support for SBD completion
evaluation of compulsory care and SBD content	
**Positive effects of the new law**	**Negative effects of the new law**
stronger emphasis on service user autonomy	no clear added value of SBDs compared to other instruments for documenting service users’ preferences
legal provisions for several instruments that provide a starting point for SBD completion	lengthy and complex legal procedures for SBD activation

### Benefits of SBDs

Participants reported various benefits of SBDs (Table 2). Most service users and professionals identified increased service user autonomy as the main benefit of having an SBD. They described SBDs as an instrument giving service users a greater say on and a more central role in the planning of their own care, because service users’ preferences are taken as a starting point for discussions with the clinical team and are then formalized into a legally binding document. The following quote illustrates the impact of SBDs on service user autonomy:

*“I believe it is part of preserving my autonomy. The moment I’m going to be assessed and everything – I am stripped of everything, my autonomy is gone… basic rights, everything is gone […]. And by deciding those things now, I still have a bit of autonomy in my own hands, my wishes – for example regarding my admission: if I am admitted, it will not be at institution X because I know too many people there.” (Participant 3 – service user)*.

Several service users and professionals indicated that SBDs contribute to an improved relationship between service users and professionals, not only because SBDs empower service users and make their relationship with clinicians more equal, but also because they provide a concrete occasion for joint discussions about service users’ preferences:

*“And this is a way in which you achieve equality and start a conversation about what is important, what is necessary. What do you want? What do you like? I think that’s worth a lot.” (Participant 5 – service user)*.

*“I think the whole procedure has been a huge boost for her and that she feels being taken very seriously and that she was given a lot of space to indicate her preferences. So it has been very good for her personally. It has also been very positive for her treatment relationship with us.” (Participant 12 – psychiatrist)*.

Many service users and professionals considered SBDs as beneficial in enabling intervention when the first signs of a mental health crisis become apparent. Early intervention can involve admission to a mental health hospital or intensified community support. Participants indicated that early intervention helps to prevent harm to service users and their social environment. They explained that SBDs can enable early intervention by authorizing compulsory care before the point is reached at which service users pose a risk of harm to self or others:

*“Now it’s much easier than if they have to wait until you are a danger to yourself or to the social environment; because I just don’t reach that level, so they wouldn’t be able to admit me.” (Participant 4 – service user)*.

*“Other options [than compulsory care] don’t work for him and he cannot be corrected in time before the danger becomes too real. So [in his SBD] he actually described a situation in which he must be treated involuntarily according to criteria that will be met before it has really reached the point of no return, in the hope that he will actually profit from the arrangement. That would not be possible without a self-binding directive, because he would not yet meet the criterion of serious disadvantage [central criterion for compulsory care under Dutch law] to be able to really proceed [with arranging compulsory care].” (Participant 11 – psychiatrist)*.

Interestingly, while the function of SBDs is to enable service users to give advance consent to compulsory care, participants indicated that SBDs can also have the effect of reducing compulsory care. While this may seem somewhat paradoxical at first sight, one participant explained that SBDs can prevent compulsory care by reminding service users of their autonomous preferences and convincing them to start or continue treatment on a voluntary basis:

*“The mere fact that they had a self-binding directive was enough. It brings them enough peace of mind to just continue treatment on an outpatient basis.” (Participant 21 – policy expert)*.

Participants thought that, in virtue of enabling early intervention in mental health crises, SBDs can have the benefit of shortening the duration of both compulsory care and recovery:

*“Before [legal provisions for SBDs existed], you first had to give someone the chance to agree with medication, then they could appeal against medication, a procedural thing. She had indicated [in her SBD] that she would like medication as soon as possible and that worked. In her case, we saw that the longer she waited with medication, the longer her recovery took, which could really extend the duration of her stay with a factor two, as did the time she needed to recover.” (Participant 18 – psychologist)*.

SBDs were also seen as rendering the experience of compulsory care less stigmatizing, traumatic, and stressful for service users because service users are actively involved in planning crisis care and are given assurance that their preferences will be respected:

*“So I think that if you can say, ‘I have drawn up a plan in which I indicate what should be done and what I prefer etc.’, it is less stigmatizing than when you have to say, ‘I have a compulsory measure that was imposed on me by the care institution due to my limited insight.’ So I really liked it.…I felt less small.” (Participant 5 – service user)*.

Professionals considered it a benefit of SBDs that they provide guidance for administering compulsory care:

*“[Her SBD describes that] if she develops manic symptoms, reduces or stops her medication, an immediate admission and immediate start with compulsory medication will follow. It also describes which medication. It’s a very concrete plan describing which interventions to use.” (Participant 18 – psychologist)*.

### Risks of SBDs

Participants also reported several risks of SBDs (Table 2). Infeasibility of SBDs emerged as the most prominent risk. Participants were concerned that decompensation may occur too quickly to be able to activate the SBD, as illustrated by this quote from a professional:

*“There is a risk that it [decompensation] sometimes goes so fast that the self-binding directive is a mere sham. Eh, so then you have made a nice plan, but then you can sometimes move from zero to one hundred instead of ten, twenty, thirty, forty …then the serious disadvantage is already so substantial that….that you have to take recourse to a crisis measure after all.” (Participant 4 – psychiatrist)*.

A further problem regarding feasibility mentioned by both service users and professionals is that the preferences of service users cannot be always followed, for example because the mental health crisis takes a course that was not anticipated or because of a scarcity of resources (e.g., the institution of choice has no beds available, or the preferred medication is unavailable):

*“So that was what we were concerned about at the time. Yes, of course she can create an ideal situation where everything will indeed go the way she wants. But you will see that the psychosis will look just a bit different or…quetiapine is not available at that time for some reason, and you still want to start olanzapine, and then everything will expire, and you just have to apply for a court order, and I think that’s a shame. […] It seemed as if a kind of sham wish list could be drafted by the patient which in the end, if it really came down to it, would actually be wiped off the table right away.” (Participant 12 – psychiatrist)*.

Participants also pointed to potential difficulties in decision-making around SBD activation. Both service users and professionals indicated that in practice it can be hard to judge whether the described circumstances of SBD activation obtain. A service user explained:

*“That’s difficult, because a bit of excitement is still okay, it says [in my SBD], and that is of course hard to estimate with my illness, because it is difficult to estimate: when is it still within normal limits and when is it becoming hypomanic?” (Participant 4 – service user)*.

Because of such uncertainties, disagreements between professionals, relatives, and service users can arise regarding the need for SBD activation. One service user gave the following example:

*“My sister once called to say that it’s not going well, and then they told her, they said, ‘We saw her yesterday and there was nothing wrong’. And I thought, ‘No’, and you know, within a few weeks, it was clear that my sister was right, my sister was there when it happened before. So it is important to me that if those things are in it, then act on it, and don’t think from your own perspective like, ‘Yes, well’.” (Participant 5 – service user)*.

Predominantly professionals raised the concern that SBDs may not be accessible during mental health crises, for instance because of limited communication between outpatient and inpatient teams. A professional recalled the following case:

*“It kind of missed its target, the outpatient team came to the ward only a week later, telling us that she had a self-binding directive. Her approach was that if she was admitted, she wanted medication immediately, but due to poor communication, this was not done for a week.” (Participant 19 – psychologist)*.

Non-compliance with SBDs, whether due to issues related to feasibility, decision-making regarding SBD activation, or inaccessibility of SBDs, can result in profound disappointment among service users and this can be detrimental to the therapeutic relationship. One service user reported that he had documented a preference for biperiden over antipsychotic medication for the treatment of psychotic episodes in his SBD. Most likely, professionals did not comply with this preference because doing so would not be in accord with professional standards (as biperiden is not an antipsychotic but a type of medication often given to reduce side-effects of antipsychotic medication, such as akathisia). Once included in an SBD, however, non-compliance with such a treatment preference can cause additional disappointment and frustration:

*“They don’t give me Akineton [brand name for biperiden] on purpose, because Akineton costs some money. Haldol [brand name for haloperidol] we have in stock… but Akineton is the key. But they don’t give me that […]. And it is clearly stated on paper that I want it that way… But I don’t even expect them to stick to it. After twenty years, I am done with them [i.e., psychiatrists].” (Participant 2 – service user)*.

Several service users and professionals worried that SBDs might not be regularly evaluated and updated. As a result, the content of SBDs may become outdated and fail to reflect service users’ preferences. One professional expressed this as follows:

*“As a practitioner in the hospital, you must be able to trust that the SBD matches the wishes of the service user. My estimation is that this is not always the case and that evaluation [of SBDs] is therefore insufficient.” (Participant 19 – psychologist)*.

### Barriers to SBD completion

Participants identified multiple barriers to SBD completion (Table 2). Both service users and professionals often mentioned that a lack of knowledge of SBDs among professionals keeps service users from completing an SBD. When professionals have not heard of SBDs or have limited knowledge of SBDs, they are unlikely to discuss the option of drafting an SBD with service users. One service user who recently drafted an SBD mentioned:

*“They made it look like it was new and… just discovered or something. And I find that shocking actually, that it has been around for so long and that the possibility has been there for so long, ouch!” (Participant 4 – service user)*.

Many service users and professionals thought that the completion rates for SBDs are low because a limited number of service users has sufficient motivation to go through the process of advance care planning and sufficient insight into their own illness. Both service users and professionals thought that insight into one’s own illness is a prerequisite for SBD completion. Service users tended to describe lack of insight as an inability to grasp the meaning and consequences of one’s own decisions rather than a denial of one’s psychiatric diagnosis. Professionals tended to think that service users often lacked sufficient insight to be able to complete an SBD without considerable support. A psychiatrist gave the following example:

*“He lacks insight into his illness to such an extent that it can conflict with how well he can overlook it all and anticipate the possibility of renewed decompensation. He can do that, overall, but to go into more detail and be able to see the importance of the preventive effect of medication, for example, that is too much.” (Participant 11 – psychiatrist)*.

Several professionals and service users thought that lack of professional support for SBD completion could explain the low uptake of SBDs. They mentioned several reasons why support may not be given, such as lack of time and professionals’ assumptions about the limited feasibility of SBDs. One service user explained:

*“Psychiatrists say that it’s so much work to draft an SBD and that it doesn’t always work well.” (Participant 1 – service user)*.

### Facilitators of SBD completion and activation

Participants also pointed to several facilitators of SBD completion and ways in which the barriers to SBD completion can be removed (Table 2). Both service users and professionals frequently mentioned professional support during the drafting process as a facilitator of SBD completion. Support tended to be understood as a close collaboration between service users and professionals in exploring potential crisis situations, service users’ preferences, and available treatment options. One role that was seen for professionals in the drafting process was checking and providing feedback on the feasibility of the preferences described in SBDs:

*“Of course, it is always the case that, because you do this together with your psychiatrist who can of course also say something like, ‘You may not want this, but we just cannot comply with that’. So of course, it is also a negotiation with that practitioner, with that psychiatrist: ‘Yes, you may want to include this, but that’s simply not possible, we cannot comply with that when push comes to shove’.” (Participant 21 – policy expert]*

Some participants mentioned the added value of involving peer experts in the drafting process. They explained that peer experts can motivate service users to complete an SBD, mediate between service users and professionals, and ensure that adequate attention is given to the perspectives of service users.

Some service users and professionals considered it important that relatives of service users be involved in the development and activation of SBDs. They explained that relatives have good insight into individual early warning signs and can therefore support service users in specifying the content of their SBD. Relatives can also signal the occurrence of early warning signs described in the SBD and hence recognize situations in which the SBD should be activated. One psychologist noted that the involvement of both a professional and a relative can put checks on the potential incompatibility of the interests of these parties, on the one hand, and those of the service user, on the other:

*“As a professional, you can ensure that potential undue influence due to power relations is checked and reflected upon. She [referring to a service user] had an intellectual disability and her partner did not. In this case, the partner and professionals have a shared responsibility to check each other’s influence on the SBD.” (Participant 18 – psychologist)*.

Service users and professionals also pointed to several facilitators of SBD activation. One of these facilitators was improving the specificity of SBD content. If an SBD contains specific information about its activation criteria and the service user’s treatment preferences, professionals get a better sense of when and how they should intervene in a mental health crisis.

Some service users and professionals had experience with the evaluation of SBDs after the provision of compulsory care, and they identified this as a facilitator of SBD activation. One service user explained that regular evaluation of the SBD helped her to specify the content of her SBD:

*“I learned more every time I got ill. So then one can include more information in one’s self-binding directive and elaborate on it, and I was able to describe several types of medication about which I could say, ‘I like those and not those’. And so I think that for new patients who start with it [drafting an SBD], it holds that you learn the more often you get ill.” (Participant 5 – service user)*.

### Effects of the new law on SBD implementation

Participants reported both positive and negative effects of the Law on Compulsory Mental Health Care on the implementation of SBDs (Table 2). Many service users and professionals indicated that the new law could facilitate the implementation of SBDs. Reflecting on their experience as a peer expert, one service user expressed it as follows:

*“We used to work very little with self-binding authorizations [the legal basis for SBD activation under the old law] and now it happens more often with the Law on Compulsory Mental Health Care. It seems to be a kind of catalyst for drafting self-binding directives with service users.” (Participant 5 – service user and peer expert)*.

Several aspects of the new law were thought to facilitate SBD completion. A first aspect mentioned by both service users and professionals was the stronger emphasis on service user autonomy. A second aspect mentioned was the law’s provision for several instruments for the documentation of service users’ preferences, such as a ‘care card’ (*zorgkaart*) and a ‘personal crisis management plan’ (*eigen plan van aanpak*). Some professionals indicated that these instruments could be used as a starting point for SBD completion. One professional put it as follows:

*“In the new law, […] you can draft a personal crisis management plan, but you can also use a care card, and I think that is an elegant way to at least encourage people to formulate their treatment wishes and needs. This is a kind of a minimal form of self-binding.” (Participant 8 – psychiatrist and medical director)*.

Participants observed that the new law also poses barriers to the implementation of SBDs. They sometimes raised the worry that professionals may not see the added value of SBDs compared to the care card and the personal crisis management plan. In contrast to SBDs, care cards have no legally binding force and personal crisis management plans are drawn up only after a mental health crisis has become apparent. Nevertheless, participants thought that the differences between these instruments might not be sufficiently clear to service users and professionals alike.

More fundamentally, participants mentioned the law’s lengthy and complex legal procedures for SBD activation as a barrier to SBD implementation. Whereas decompensation can occur and the need for SBD activation can arise within a couple of days, the new legal procedure for obtaining authorization of compulsory care based on an SBD takes much longer than that. Participants offered this as an explanation for why professionals are reluctant to provide support for SBD completion and tend to resort to other ways of considering service users’ preferences in providing compulsory care. A professional explained how experience with legal procedures resulted in low support for SBD implementation:

*“I don’t see the advantage [of SBDs] yet, and there is also this very annoying thing, eh, that … at the court they are not all used to it yet and you can see that processes are very slow. So whereas supposedly that authorization should be granted very quickly, it did not work for that patient of mine, for example. I had consulted with him, and everyone including the court agreed that it had to be done now. Then it should be possible to obtain authorization within three days. Well, that didn’t happen.” (Participant 16 – nurse practitioner)*.

## Discussion

Stakeholders with personal or professional experience with legally enforceable SBDs reported multiple opportunities and challenges of SBDs, including benefits and risks of SBDs and barriers and facilitators related to their implementation. Stakeholders’ views on the effects of the new law on the implementation of SBDs were mixed.

Stakeholders with personal or professional experience with SBDs confirmed some of the benefits that have been associated with SBDs in the conceptual literature, such as the promotion of service user autonomy, the improvement of the therapeutical relationship, and the facilitation of early intervention [5–7, 10–14]. By contrast, they did not express the fundamental ethical concerns regarding SBDs which have been articulated by several ethicists and legal scholars, such as an increase of coercion, the impossibility to accomodate for changes of mind, and expired consent [3, 4, 15, 16], suggesting that these fundamental ethical concerns do not apply in practice. They instead focused on practical and logistical challenges. Participants in our study tended to endorse SBDs because they thought that the benefits of SBDs outweigh the risks and that the practical and logistical challenges can be addressed in policy-making and clinical implementation.

The high endorsement of SBDs among participants should be interpreted with caution due to the likelihood of selection bias. Because we included service users who have an SBD and professionals who supported service users in drafting an SBD, participants were likely to endorse SBDs overall. That said, it is nevertheless significant that stakeholders with personal or professional experience with legally enforceable SBDs did not voice fundamental ethical concerns about the instrument.

Our findings on the opportunities and challenges of SBDs confirm findings from qualitative stakeholder studies conducted in contexts where SBDs are not legally enforceable [22, 23, 27]. Accordingly, there seem to be no large differences between the perspectives of stakeholders who consider SBDs hypothetically and the perspectives of stakeholders with personal or professional experience with legally enforceable SBDs. While this seems to suggest that the potential benefits of SBDs materialize in clinical practice and that no unexpected challenges occur, more research is needed to make definite statements on this.

Our findings on the barriers to and facilitators of SBD implementation largely overlap with the findings on the barriers and facilitators of the implementation of psychiatric advance directives without self-binding clause [9, 17]. The implementation issues regarding SBDs thus seem not to go beyond those regarding psychiatric advance directives. This suggests that the implementation of SBDs can be promoted by raising awareness among service users and professionals, creating a technical infrastructure ensuring SBD accessibility, developing training modules for professionals, offering professional support for SBD completion, involving persons of trust in SBD completion and activation, and developing guidance for service users in the form of SBD templates and resource materials [22, 28, 29].

One barrier to SBD implementation which we found in this study is particular to the Dutch context, namely the lengthy and complex procedures for obtaining legal approval for SBD activation. Members of our research team anticipated this barrier from an ethico-legal perspective [8]. Findings from the current study indicate that the procedure for obtaining legal authorization of compulsory care based on an SBD should not constitute a barrier to early intervention and hence should be shorter than the mean time in which decompensation occurs. To this end, a special mechanism for legal authorization of compulsory care based on an SBD can be created, as members of our research team have proposed elsewhere [8]. According to this mechanism, the service user’s person of trust and treating psychiatrist can jointly submit a request for SBD activation, upon which the judge hears the service user and grants or denies authorization of compulsory care within 24 h. This mechanism is compatible with the findings from the current study and could inform SBD policy in other jurisdictions as well.

### Strengths and limitations

To the best of our knowledge, this is the first study on SBDs worldwide with stakeholders who have personal or professional experience with legally enforceable SBDs. A limitation of the study is that despite great effort, we were able to include only a relatively small number of service users in the study. This was due to the inclusion criteria of our study and the very low completion rates for SBDs in the Netherlands. The generalizability of results is limited by the fact that service users with an SBD and professionals working with service users who have SBDs may have more positive views on SBDs than other service users and professionals, in particular those who have decided against using SBDs. Three of the service users included in our study worked as peer expert in a mental health institution, on account of which they may have more positive attitudes toward the mental health system in general and SBDs in particular. Quantitative surveys in representative populations are needed to get a more accurate view of stakeholders’ perspectives on the opportunities and challenges of SBDs.

## Conclusions and clinical recommendations

Stakeholders who have personal or professional experience with legally enforceable SBDs endorse SBDs and advocate further clinical implementation of the instrument. They consider SBDs as having major benefits to service users and other stakeholders and tend not to express fundamental ethical concerns about the instrument. They do perceive SBDs as posing various practical and logistical challenges. The following recommendations can help professionals to address these challenges:

- Raise awareness about SBDs.
- Develop guidance on SBD completion.
- Provide support and give relevant medical information during SBD completion.
- Involve a person of trust in SBD completion and activation.
- Ensure accessibility of SBDs in mental health crises.
- Comply with SBD instructions, unless this would violate legal regulations or professional standards.
- Evaluate compulsory care in light of SBD instructions jointly with service users and persons of trust.
- Update SBDs based on the results of the evaluation of compulsory care.

## Electronic supplementary material

Below is the link to the electronic supplementary material.

Additional File 1: Interview guide version for service users

## Data Availability

An English translation of the original Dutch interview guide is available in the online supplementary materials. It is not possible to share the research data publicly as this may compromise the privacy of research participants. The anonymized data are available from the corresponding author upon reasonable request.

## List of abbreviations

SBD: self-binding directive
