# Supplementary material for: Opportunities and challenges of self-binding directives: an interview study with mental health service users and professionals in the Netherlands
Source: BMC Med Ethics. 2023 Jun 3;24:38. doi: 10.1186/s12910-023-00915-y (PMC10239595; doi:10.1186/s12910-023-00915-y)
Supplement: Supplementary file 1 — Additional File 1: Interview guide version for service users [file 12910_2023_915_MOESM1_ESM.docx]

**Interview guide – version for service users**

Opportunities and challenges of self-binding directives

Translated from the Dutch original.

1. **Procedure**
2. **Information about self-binding directives**

- Are you familiar with self-binding directives? Do you have a self-binding directive? Or do you know someone who has one?
- Through whom or through which authority did you hear about the possibility to draft a self-binding directive?
- Do you have experience with crisis cards or another type of psychiatric advance directive?
- What conditions (as far as you know) must be met for a self-binding directive to be drawn up?

1. **Drafting a self-binding directive**

- What motivated you to (not) draw up a self-binding directive?
  - Do you have experiences with compulsory admission or treatment?
  - What do you hope to achieve with a self-binding directive?
- How did drafting the self-binding directive work? Who took the initiative?
- Who was involved in drafting the self-binding directive?
  - What role did relatives play in this?
  - Who do you think should be involved in this?
- In your opinion, did you receive sufficient support in drawing up your self-binding directive?
  - Do you have points for improvement?
  - What kind of support is desirable?
- Expectations:
  - What were your expectations regarding self-binding directives?
  - Have mutual expectations been discussed during the drafting process?
  - Have the circumstances in which the self-binding directive would apply been explicitly discussed with you during the drafting process?
- What do you think is the most important content of your self-binding directive?
- Was a care plan also drawn up at the time when you completed the self-binding directive? Was/would that be helpful?
- What barriers have you encountered in drafting your self-binding directive?
- Do you think the procedures for drawing up a self-binding directive are clear?
- Do you think everyone should be able to draft a self-binding directive, or is it not suitable for everyone?

1. **Storing and updating self-binding directives**

- Who in your social environment is aware of your self-binding directive?
- With the introduction of the new Law on Compulsory Mental Health Care (Wvggz), you can now determine the period of validity of your self-binding directive yourself. The term used to be 1 year under the old law (BOPZ). What do you think of this change?
- Do you regularly update your self-binding directive regularly? At which times do you think this is desirable?

1. **Application of self-binding directives**

- Have you experienced your self-binding directive being applied? If so, do you have experience with being compulsorily admitted or treated based on your self-binding directive? Was this different from prior compulsory admissions or treatments without self-binding directive?
- In which situations do you consider the application of your self-binding directive to be appropriate? Can you give concrete examples?
- In which situations would the application of a self-binding directive not be useful or appropriate? Can you give concrete examples?
- In your opinion, is it usually possible to intervene in a mental health crisis in time with the use of a self-binding directive? What is the difference with situations in which one does not have a self-binding directive?
- Has your self-binding directive always been followed? If not, what were the stated reasons for not following the directive?

1. **Debriefing**

- Has your self-binding directive been evaluated after compulsory care based on the directive has been provided? If yes, how?
- (Imagine) you have been compulsorily admitted on the basis of your self-binding directive; would you like this to be discussed with you afterwards?
- Have you adjusted the content of your self-binding directive based on your experiences? Why/why not?
- Who was involved in debriefing (if it took place)? Who should be involved in a debriefing?

1. **Opportunities, risks, and regulation**
2. **Opportunities of self-binding directives**

- How do you experience the opportunities for drawing up self-binding directives in the Netherlands?
- What added value do self-binding directives have in your opinion?
- What do you think is the effect of self-binding directives on the use of coercion in mental health care?
- What do you think are the effects of a self-binding directive on the course of a mental health crisis?
- What do you think are the effects of self-binding directives on recovery and reintegration, compared to situations in which one does not have a self-binding directive?

1. **Risks of self-binding directives**

- Have you ever encountered problems with your self-binding directive? What problems were these?
- In what situations could self-binding directives lead to problems? Can you mention concrete examples?

1. **Monitoring and due care criteria**

- Suppose that during a future mental health crisis you do not want to be treated according to your self-binding directive: Who do you think should decide whether compulsory admission or treatment can take place on the basis of your self-binding directive?
- In your opinion, how could any risks of self-binding directives be avoided?
- How much value do you place on the fact that all steps of the procedure are followed carefully compared to receiving care as soon as possible?
- You can amend or withdraw your self-binding directive in the interim. What conditions do you think should be met for this to be possible?

1. **Uptake**

- Self-binding directives are rarely used until now. Do you have an explanation for that?
- Through which channels would information about self-binding directive best be disseminated?
  - What would be the roles of: psychiatrists, MIND [service user organization], and PVP [service user representative organization] respectively?

1. **Wvggz**

- How informed are you about current changes in the practice of self-binding directives?
- Do you feel that you have been informed well about the changes?
- Have you drawn up a self-binding directive after the new law entered into force? Why/why not?
- What do you think the impact of the new law will be on the use of self-binding directives? Do you think they will be used more/less often? Will it be easier or harder to use them?
- In the new law, service users from the age of 12 can draw up a self-binding directive jointly with their representative and the treating mental health professional. What do you think of this new age limit of 12 years?

1. **Final questions**

- Would you recommend drafting a self-binding directive to others? Why/why not?
- What would you like to say to others who are interested in drafting a self-binding directive?

**Interview guide – version for professionals**

Opportunities and challenges of self-binding directives

Translated from the Dutch original.

1. **Procedure**
2. **Information about self-binding directives**

- Have you been involved in the drafting of self-binding directive? Do you treat service users who have a self-binding directive?
- Do you have experience with crisis cards or another type of psychiatric advance directive? How do self-binding directives relate to these?
- What conditions must be met (as far as you know) for drafting a self-binding directive? What are the most important conditions that you consider when service users want to draw up a self-binding directive?

1. **Drafting a self-binding directive**

- What motivated you to insist/not to insist on the use of self-binding directives?
  - What do you hope to achieve with self-binding directives?
- How did drafting the self-binding directive work? Who took the initiative?
- Who was involved in drafting the self-binding directive?
  - What role did relatives play in this?
  - Who do you think should be involved in this?
- In your opinion, do clients receive sufficient support in drawing up self-binding directives?
  - Do you have points for improvement?
  - What kind of support is desirable?
- Expectations:
  - What were your expectations regarding self-binding directives?
  - Have you explicitly considered the circumstances in which the self-binding directive would apply during the drafting process?
- What do you think is the most important content of a self-binding directive?
- Was a care plan also drawn up at the time when you completed the self-binding directive? Was/would that be helpful?
- What barriers have you encountered in drafting self-binding directives?
- Do you think the procedures for drawing up a self-binding directive are clear?
- Do you think everyone should be able to draft a self-binding directive, or is it not suitable for everyone?

1. **Storing and updating the self-binding directive**

- With the introduction of the new Law on Compulsory Mental Health Care (Wvggz), the period of validity of self-binding directives can now be determined by service users themselves. The term used to be 1 year under the old law (BOPZ). What do you think of this change?
- Are self-binding directives regularly updated? At which times do you think updates are desirable?

1. **Application of self-binding directives**

- Have you experienced a service users’ self-binding directive being applied? If so, do you have experience with the process of arranging compulsory admission or treatment based on a self-binding directive? Was this different from compulsory admissions or treatments without self-binding directive?
- In which situations do you consider the application of a self-binding directive? Can you give concrete examples?
- In which situations would the application of a self-binding directive not be useful or appropriate? Can you give concrete examples?
- In your opinion, is it usually possible to intervene in a mental health crisis in time with the use of a self-binding directive? What is the difference with situations in which service users do not have a self-binding directive?
- Are service users who have an SBD also sometimes involuntarily admitted to hospital based on a crisis measure?
- In your experience, are self-binding directives always followed? If not, what were the reasons for not following the directive?

1. **Debriefing**

- Is the provision of compulsory care based on a self-binding directive retrospectively evaluated? If yes, how?
- In your experience, is the content of self-binding directives adjusted based on experience? Why/why not?
- Who should be involved in debriefing?

1. **Opportunities, risks, and regulation**
2. **Opportunities of self-binding directives**

- How do you experience the opportunities for drawing up self-binding directives in the Netherlands?
- What added value do self-binding directives have in your opinion?
- What do you think is the effect of self-binding directives on the use of coercion in mental health care?
- What do you think are the effects of a self-binding directive on the course of a mental health crisis?
- What do you think are the effects of self-binding directives on recovery and reintegration, compared to situations in which service users do not have a self-binding directive?

1. **Risks of self-binding directives**

- Have you ever encountered problems with a service user’s self-binding directive? What problems were these?
- In what situations could self-binding directives lead to problems? Can you mention concrete examples?

1. **Monitoring and due care criteria**

- Suppose a client does not want to be treated according to their self-binding directive during a mental health crisis: Who do you think should decide whether compulsory admission or treatment can take place on the basis of the self-binding directive?
- In your opinion, how could any risks of self-binding directives be avoided?
- How much value do you place on the fact that all steps of the procedure are followed carefully in light of the idea that service users should receive care as soon as possible?
- Service users can amend or withdraw their self-binding directive in the interim. What conditions do you think should be met for this to be possible?

1. **Uptake**

- Self-binding directives are rarely used until now. Do you have an explanation for that?
- Through which channels would information about self-binding directive best be disseminated?
  - What would be the roles of: psychiatrists, MIND [service user organization], and PVP [service user representative organization] respectively?

1. **Wvggz**

- What do you notice any changes in the use of self-binding directives after the introduction of the Wvggz?
- What works under the new law? And what doesn’t work?
- Do you feel that you have been informed well about the changes?
- Have you drawn up self-binding directives after the Wvggz entered into force? Why/why not?
- What do you think the impact of the new law will be on the use of self-binding directives? Do you think they will be used more/less often? Will it be easier or harder to use them?
- In the new law, service users from the age of 12 can draw up a self-binding directive jointly with their representative and the treating mental health professional. What do you think of this new age limit of 12 years?

1. **Final questions**

- Would you recommend drafting a self-binding directive to others? Why/why not?
- What would you like to say to others who are interested in drafting a self-binding directive?
